# The relative impact of socioeconomic position and frailty varies by population setting

**DOI:** 10.1002/agm2.12200

**Published:** 2022-02-27

**Authors:** Elliot Goodyer, Jasmine C. Mah, Apoorva Rangan, Petronella Chitalu, Melissa K. Andrew, Samuel D. Searle, Daniel Davis, Alex Tsui

**Affiliations:** 1 MRC Unit for Lifelong Health and Ageing at UCL, London, UK; 2 Department of Medicine, Dalhousie University, Halifax, Nova Scotia, Canada; 3 School of Medicine, Stanford University, Stanford, CA, USA; 4 Division of Geriatric Medicine, Dalhousie University, Halifax, Nova Scotia, Canada; 5 Department of Medicine for the Elderly, University College Hospital, London, UK

**Keywords:** acute hospitalisation, epidemiology, frailty, mortality, socioeconomic position

## Abstract

**Introduction:**

Frailty and socioeconomic position (SEP) are well-established determinants of health. However, we know less about the contributions of frailty and SEP in older adults, especially in acute settings. We set out to answer how frailty and SEP might influence health outcomes in older people, comparing a population sample and patients managed by a speciality acute frailty service.

**Methods:**

We used the Delirium and Population Health Informatics Cohort, a population sample of 1510 individuals aged ≥70 years from the London Borough of Camden and 1750 acute frailty patients. SEP was determined using the Index of Multiple Deprivation. Linear and Cox proportional hazard regression models were conducted to assess SEP on frailty, readmission, and mortality outcomes.

**Results:**

In the population sample, SEP was significantly associated with frailty and mortality with successive increases in rate of death for each IMD quintile (HR = 1.28, 95% CI 1.11 to 1.49, *P* < 0.005). Increasing SEP, age, and admission status among hospitalized individuals were associated with greater frailty. For individuals seen by the speciality frailty service, SEP was not associated with frailty, mortality, or readmission.

**Discussion:**

When older people experience acute illness severe enough to require secondary care, particularly specialist services, this overcomes any prior advantages conferred by a higher SEP.

## 1 Introduction

Frailty and socioeconomic position (SEP) are two well-established determinants of health in older adults. In community-dwelling older adults, frailty is consistently associated with a higher likelihood of death,^1,2^ worsening disability,^1,3^ recurrence of falls,^1,4^ increased hospitalization,^1^ and nursing home admission.^1,5^ Social inequalities include differences in income, wealth, housing security, education, and occupation, among others; these similarly contribute to adverse health outcomes.^6,7^

For older adults, social inequalities accumulate over the life course leading to relative disparities in socioeconomic position, with profound impacts on lifespan, health, and well-being.^8,9^ Longitudinal studies have also demonstrated consistent relationships between socioeconomic position and frailty.^10^ In the Survey of Health, Ageing and Retirement in Europe, a participant with the lowest levels of education, occupation, income, and wealth was as frail as a participant 7 years older with the highest levels of these measures.^11^ In older adults, lower socioeconomic position is associated with more frequent episodes of acute health problems leading to deterioration and instability in baseline frailty and increased mortality.^12^ However, the extent to which these relationships hold true across different settings within health and social care systems is less clear. For example, how much does SEP continue to affect clinical outcomes once an older person is admitted to hospital? Quantifying these effects might have implications for assessing older people with acute illness at the individual level, as well as the service design at the population level.

**Figure 1:**
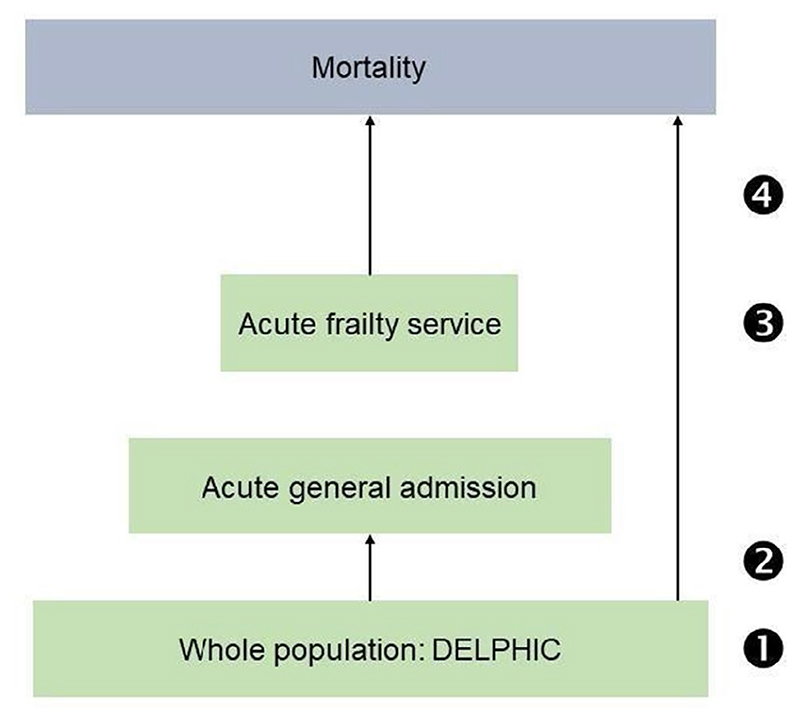
Schema showing inter-relationship of datasets. Questions: What is the relationship between SEP and frailty in: 1. a population sample? 2. those acutely admitted to hospital? 3. those seen by a specialist frailty service? 4. What is the relationship between SEP and mortality in a population, compared with specialist frailty service?

To address the question of how frailty and SEP might influence outcomes in older people, we used overlapping prospective clinical and population data. We hypothesized that lower socioeconomic position might be associated with more frailty and mortality. We addressed these by focusing on four specific questions (Figure 1): What is the relationship between SEP and frailty in: 1. a population sample? 2. those acutely admitted to hospital? 3. those seen by a specialist frailty service? 4. What is the relationship between SEP and mortality in a population, compared with a specialist frailty service? While there are different priorities for different settings— public health approach for addressing health inequality, and a direct clinical one in the acute context—our aim in these analyses was to describe the points at which these two factors transition within a defined geographic health service.

## 2 Methods

### 2.1 Data sources

#### Population sample

DELPHIC is an ongoing population-representative study, following individuals aged ≥70 years from the London Borough of Camden. Its primary purpose is to undertake longitudinal assessments across community and acute hospital settings, focusing on measures often unreliably coded in electronic health records (e.g. cognition, physical function, frailty). University College Hospital, London, UK, is one of two acute hospitals in Camden (see below). Full details of the study have previously been reported.^13,14^ In brief, the sample was mainly enrolled from primary care lists and is representative of the borough in terms of age distribution and income deprivation indices. We assessed participants through telephone interviews and on each hospital admission. In addition, researchers had access to all health and social care data to corroborate clinical information. The data presented here are an analysis of the first 1510 participants recruited between 2017 and 2019.

*Acute frailty service (specialist hospitalized sample)*: We obtained data on unscheduled admissions to a single tertiary hospital (University College Hospital) between April 2015 and January 2017, before the establishment of DELPHIC. Here, an acute frailty service proactively managed all older frail patients identified through screening referrals to general internal medicine.^15^ During the first 24 h of each consecutive admission, a specialist geriatrician assessed for dementia, delirium, falls (history), and frailty. We collected demographic data for each participant (age, sex, ethnicity, postcode). We excluded patients who lived in any form of long-term care (assisted/ nursing level care) because neighborhood indices of deprivation are less reliable in this context.

### 2.2 Outcome measures

The outcomes of interest were current frailty (*Questions 1*, *2*, and *3*), and subsequent readmission and mortality (*Question 4*). In the population sample, we derived a 35-item Frailty Index representing the proportion of accumulated health deficits (0 to 1) in the population sample. Items used included: self-rated health, comorbidities, sensory difficulties (including vision and hearing), incontinence, falls, mobility, personal and instrumental activities of daily living, polypharmacy, cognitive function, and quality of life. The Frailty Index was calculated using standard procedures.^16^ In the acute frailty service cohort, frailty was measured using the Clinical Frailty Scale (CFS), referring to a period of health 2 weeks before acute presentation. The CFS is a 9-point score where 1 represents robust and active people and 9 are those approaching the end of life.

For *Question 4*, we considered any hospitalization (population sample) or readmissions (acute frailty service). We determined mortality through notifications to the NHS Spine, a statutory register of all deaths in England. We considered all-cause mortality from the date of admission to hospital to December 2018 (44 months).

### 2.3 Exposures

For each patient postcode (population sample and acute frailty service), we determined the Index of Multiple Deprivation (IMD) using the 2019 English Indices of Deprivation. IMD is an ecological measure of overall deprivation calculated for each Lower-layer Super Output Area in England. Lower-layer Super Output Areas represent neighborhoods with population sizes between 1200 and 3000. IMD is derived from 37 separate indicators organized across seven sub-domains: income, employment, health, crime, education, barriers to housing and services, and living environment. We used the IMD decile rank for individuals’ Lower-layer Super Output Area, where lower ranks have higher levels of deprivation. A history of falls in the previous year, delirium on presentation to hospital, and dementia were operationalized as binary values (yes/no).

In the population sample (DELPHIC), we used educational attainment and occupational class as measures of individual SEP. Educational attainment was defined as having completed primary, secondary, or tertiary education (three categories). Occupational class was derived from the Office for National Statistics’ UK Occupational Skill Classification. Level 1 and 2 include skills from compulsory education and post-compulsory education. Level 3 and 4 refer to skills from additional work-related training (normally without a bachelor’s degree) and professional skills with a degree or equivalent.

### 2.4 Statistical analysis

#### 2.4.1 Socioeconomic position and frailty

*Question 1* and *2*: The frailty index was the primary outcome, with educational attainment (three levels), occupational class (four levels), IMD or income deprivation affecting older people index (centile rank), and hospitalization status (yes/no) estimated in linear regression models adjusted for age and sex.

*Question 3*: We used linear regression to estimate the relationship between CFS score (as a continuous measure) and IMD decile, adjusted by age (in years), sex, and presence of dementia.

#### 2.4.2 Socioeconomic position, mortality and readmission

*Question 4*: We performed Cox proportional hazards regression to quantify the association between IMD decile and survival and readmission, adjusting for the same covariates (age, sex, and dementia) in addition to the presence of delirium and a history of falls within the last 12 months. We tested for interactions between SEP and admission status to assess if the relationship between SEP morality was different in those hospitalized.

All analyses were undertaken using Stata (version 16.1 and Python 3.7.6).

## 3 Results

### 3.1 Population sample

The average age was 78 (SD 16.7) years and 57% were women. Frailer participants were older and more likely to have been admitted to hospital at least once. Educational attainment was high in this cohort, with 1092 (72%) having at least a bachelor’s degree. This group showed the lowest levels of frailty (lowest education *n* = 521, highest education *n* = 220, *P* < 0.01). Occupational class followed a similar distribution (Table 1).

*Question 1*: Neighborhood deprivation was associated with frailty, on both measures of disadvantage: index of multiple deprivation (IMD) (mean score 15.5 in frailest tertile, 19.0 in the least frail tertile, *P* < 0.01) and income deprivation of older adults (IDO) (mean score 0.18 in frailest tertile, 0.23 in the least frail tertile, *P* = 0.04) (Table 1).

**Figure 2:**
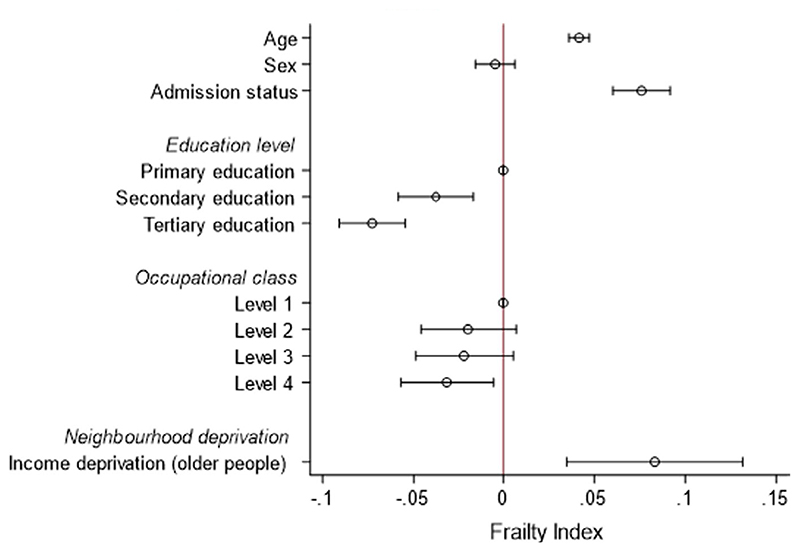
The relationship between socioeconomic position and frailty in a population sample (Question 1)

**Figure 3:**
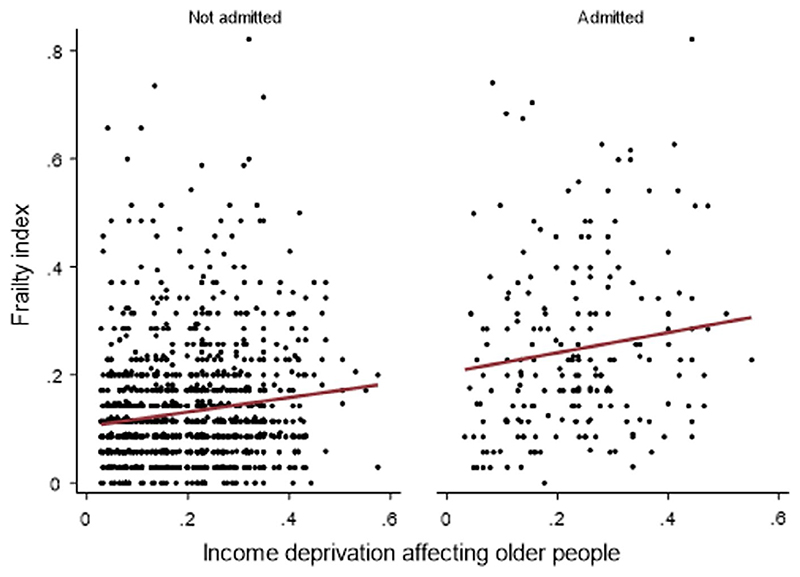
The relationship between SEP and frailty in a population sample, stratified by admission status (Question 2)

*Question* *2*: In multivariable models, age (β = 0.04; 95% CI 0.04 to 0.05), admission status (β = 0.08; 95% CI 0.06 to 0.09) and IDO (β = 0.08; 95% CI 0.03 to 0.13) were associated with frailty (Figure 2, Supplementary Table S1). The inverse association between educational attainment and frailty persisted, but this was not the case for occupational class (Figure 1). We did not demonstrate any interaction between IDO and admission status (*P* > 0.05); the degree of the relationship was the same regardless of all-cause hospitalization (Figure 3).

### 3.2 Acute frailty service

Among 2259 admissions in 1750 individuals, mean age was 85 (SD 6.8) years and 57% were women (Table 2). The lowest two IMD quintiles accounted for 63% of admissions; <1% were in the most advantaged quintile.

**Figure 4:**
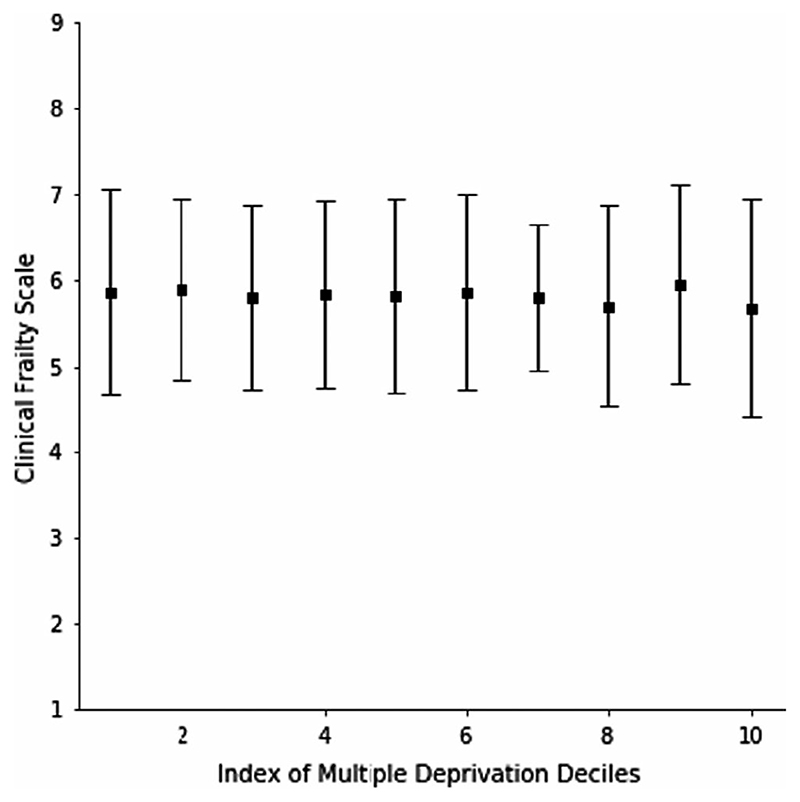
The relationship between socioeconomic position and frailty presenting to an acute frailty service (Question 3)

*Question 3*: In a multivariable model, age was associated with CFS score (β = 0.02; 95% CI 0.01 to 0.03, *P* < 0.01), as was dementia (β = 0.66; 95% CI 0.58 to 0.75, *P* < 0.01), but not sex (β = 0.07; 95% CI -0.03 to 0.17, *P* = 0.15). IMD decile was not associated with frailty (β = −0.01; 95% CI −0.03 to 0.02) (Figures 3 and 4).

**Figure 5:**
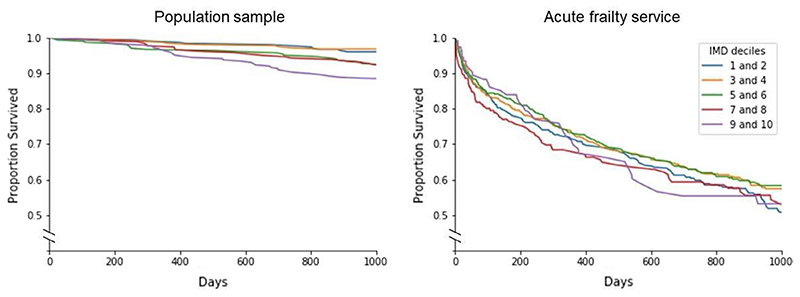
The relationship between socioeconomic position and mortality (Question 4)

*Question 4*: In the population cohort, SEP was associated with mortality, with successive increases in rate of death for each IMD quintile (HR = 1.28, 95% CI 1.11 to 1.49, *P* < 0.005), even after adjustment for age, sex, frailty, delirium, and falls history (Table 3). However, in the Acute Frailty Service cohort, IMD decile was neither associated with mortality (HR = 1.00, 95% CI 0.96 to 1.03 *P* = 0.82) nor readmission (HR = 0.95, 95% CI 0.90 to 1.00, *P* = 0.06) in multivariable analysis. Age, sex, and CFS were associated factors for mortality, with similar patterns observed for risk of readmission (Table 3) (Figure 5).

## 4 Discussion

We showed different relationships between SEP and frailty in overlapping population and specialist clinical samples. In a population cohort, there were clear associations between SEP and frailty. Frailty scores were higher in those hospitalized, but the gradient of the SEP–frailty relationship was consistent. Though patients with high SEP were much less likely to present to the Acute Frailty Service, those subsequently being managed by this service did not vary by degree of SEP. Taken together, our findings suggest that by the time patients were selected into a specialist service, SEP was no longer a driving part of ill health, and frailty-specific factors may predominate.

Our data should be treated with caution. Although we had individual-level measures of SEP in the population cohort, IMD was the main SEP measure. As an ecological variable, neighborhood factors may not directly represent an individual’s SEP. It is also possible that the Lower-layer Super Output Areas used to define IMD are more heterogeneous in London due to the density and variability of different housing types in close proximity. Our findings are based on a single site. In keeping with other observational data, the associations demonstrated may be subject to residual confounding. Where we did account for confounders, they varied in their availability and operationalization. The samples differed in clinical characteristics by virtue of their setting. Despite this, we have had the advantage of complementary datasets, where a specialist referral service was nested within a population-representative sample, with reliable ascertainment of frailty and mortality outcomes.

The broader findings are consistent with previous studies showing a clear relationship between SEP and frailty, with comparable effect sizes to other community studies.^17^ However, our results from the specialist frailty cohort have not previously been shown.

A scoping review investigating frailty in the acute setting demonstrated associations with mortality, increased length of stay, and institutionalization post-discharge.^18^ Most other studies of SEP are in community-based populations^19–21^; socioeconomic position is rarely assessed in acutely hospitalized patients, and even less so in older adults. For example, while an association between social deprivation and in-hospital mortality has been reported in critical care, this did not extend to complications (acute kidney injury or ICU admission), length of stay or readmissions.^22^ Increased social vulnerability is predictive of long-term care outcomes in hospitalized older adults, but only for the oldest-old with lower levels of frailty.^23^

What accounts for the lack of association between IMD and frailty in the specialist cohort? It would seem that the social determinants of health operate at each level (population, general admission) until patients are selected into a specialist frailty service. Once managed in this setting, SEP no longer appears to drive mortality or readmission. This may be specific to a publicly funded health system able to counter the socioeconomic position health gradient, and international comparisons would be needed to confirm this. On the other hand, it is possible that the specialist cohort are so frail that any proximal effects from SEP no longer come into play. To examine this, future studies may look at conducting analysis in a group of patients where SEP is less accounted for in care plans, for example in a surgical setting.

Our study demonstrates that illness severe enough to require secondary care, particularly specialist services, overcomes prior advantages conferred by a higher SEP. Though policies that aim to reduce socioeconomic inequalities may be of benefit at the population level, acute frailty services in health systems comparable to the UK are likely to provide the same benefit to individuals across the spectrum of socioeconomic advantage.

## Supplementary Material

Additional supporting information may be found in the online version of the article at the publisher’s website.

Supporting Information

## Figures and Tables

**Table 1:** Descriptive characteristics of the population cohort (DELPHIC), showing increased gradient in deprivation across tertiles of frailty

	Least frail^a^		Medium frail^a^		Most frail^a^		
	*N*	Mean (SD) or %	*N*	Mean (SD) or %	*N*	Mean (SD) or %	*p*
Age	612	75 (4.8)	459	78 (5.5)	437	82 (6.4)	<0.01
Sex	364	61	251	56	220	53	0.04
Admission status	35	6	48	10	125	14	<0.01
Educational attainment							<0.01
Primary	27	4	50	11	136	32	
Secondary	60	10	56	12	71	17	
Tertiary	521	86	351	77	220	52	
Occupational class^b^							<0.01
Level 1	13	2	15	3	53	12	
Level 2	65	11	73	16	99	23	
Level 3	91	15	66	14	89	21	
Level 4	437	72	304	66	192	44	
Index of multiple deprivation	595	15.5 (8.3)	444	16.0 (9.0)	415	19.0 (9.9)	<0.01
Income deprivation^c^ (older adults)	595	0.18 (0.1)	444	0.19 (0.1)	415	0.23 (0.2)	0.04

a Frailty categories determined by tertiles of Frailty Index score.

b Office for National Statistics occupational skills classification.

c Income deprivation affecting older adults index.

**Table 2:** Study population stratified by quintiles of Index of Multiple Deprivation

			Index of Multiple Deprivation by Quintile					
	Overall	Missing	1^st^	2^nd^	3^rd^	4^th^	5^th^	*P*
N (%)	2259		583 (26)	845 (37)	507 (22)	205 (9)	89 (<1%)	<0.01
Age (%)	85.0 (7)	0	83.7 (7)	85.2 (7)	86.0 (7)	84.8 (6)	85.7 (6)	<0.01
Sex (%)	1276 (57)	0	333 (57)	468 (55)	297 (59)	110 (54)	55 (62)	0.55
Dementia (%)	1081 (48)	3	331 (57)	390 (46)	211 (42)	101 (49)	35 (39)	<0.01
Delirium (%)	713 (32)	0	207 (36)	251 (30)	156 (31)	68 (33)	22 (25)	0.01
Falls (%)	1016 (45)	2	251 (43)	394 (47)	226 (45)	89 (43)	40 (45)	0.73
Clinical Frailty Score (%)	5.8 (1)	3	5.9 (1)	5.8 (1)	5.8 (1)	5.7 (1)	5.9 (1)	0.34
Died (%)	773 (34)	0	203 (35)	279 (33)	167 (33)	79 (38)	34 (38)	0.50
Readmission (%)	684 (33)	170	194 (36)	259 (33)	150 (32)	51 (28)	25 (31)	0.29

**Table 3:** Population and specialist samples showing relationship between mortality and readmission

	Population sample			Specialist sample					
	Mortality			Mortality			Readmission		
	HR	95% CI	*P*	HR	95% CI	*P*	HR	95% CI	*P*
Age (per year)	1.07	1.04 to 1.11	<0.005	1.04	1.03 to 1.05	<0.005	1.03	1.01 to 1.04	<0.01
Sex	0.61	0.41 to 0.91	0.01	0.68	0.59 to 0.78	<0.005	0.87	0.71 to 1.07	0.15
Frailty Index^a^	1.56	1.38 to 1.76	<0.005	-	-	-	-	-	-
Clinical Frailty Scale	-	-	-	1.44	1.33 to 1.56	<0.005	1.31	1.20 to 1.42	<0.01
Falls (History)	1.05	0.66 to 1.65	0.84	0.84	0.73 to 0.97	0.02	1.00	0.83 to 1.20	0.97
Delirium	0.84	0.48 to 1.46	0.53	1.4	1.2 to 1.63	<0.005	1.01	0.87 to 1.18	0.88
Dementia	-	-	-	0.87	0.74 to 1.02	0.08	0.75	0.62 to 0.91	<0.01
IMD Decile	1.28	1.11 to 1.49	<0.005	1	0.96 to 1.03	0.82	0.95	0.90 to 1.00	0.06

a Represents 10% increase or 3.5 more deficits.
