# Supplementary material for: The relative impact of socioeconomic position and frailty varies by population setting
Source: Aging Med (Milton). Author manuscript; Available in PMC 2022 Mar 14. (PMC8917265; doi:10.1002/agm2.12200)
Supplement: Supporting Information [file EMS143781-supplement-Supporting_Information.docx]

**Supporting information**

**Supplementary table 1** Descriptive characteristics of the population cohort (DELPHIC), showing relationship with frailty index in a univariable and multivariable analysis

|  | | **Univariable** | | | |  | **Multivariable** | | | |
| --- | --- | --- | --- | --- | --- | --- | --- | --- | --- | --- |
|  | | **β** | **95% CI** | | **p** |  | **β** | **95% CI** | | **p** |
| Age (per SD) | | 0.06 | 0.05 | 0.06 | <0.01 |  | 0.04 | 0.04 | 0.05 | <0.01 |
| Sex | | -0.01 | -0.02 | 0.00 | 0.19 |  | 0.00 | -0.02 | 0.01 | 0.42 |
| Admission status | | 0.12 | 0.10 | 0.13 | <0.01 |  | 0.08 | 0.06 | 0.09 | <0.01 |
|  | |  |  |  |  |  |  |  |  |  |
| Educational attainment | |  |  |  | <0.01 |  |  |  |  | <0.01 |
|  | Primary | [ref] |  |  |  |  | [ref] |  |  |  |
|  | Secondary | -0.08 | -0.11 | -0.06 |  |  | -0.04 | -0.06 | -0.02 |  |
|  | Tertiary | -0.14 | -0.15 | -0.12 |  |  | -0.07 | -0.09 | -0.05 |  |
|  | |  |  |  |  |  |  |  |  |  |
| Occupational class^†^ | |  |  |  | <0.01 |  |  |  |  | 0.09 |
|  | Level 1 | [ref] |  |  |  |  | [ref] |  |  |  |
|  | Level 2 | -0.05 | -0.08 | -0.02 |  |  | -0.02 | -0.05 | 0.01 |  |
|  | Level 3 | -0.08 | -0.11 | -0.05 |  |  | -0.02 | -0.05 | 0.01 |  |
|  | Level 4 | -0.12 | -0.15 | -0.09 |  |  | -0.03 | -0.06 | -0.01 |  |
|  | |  |  |  |  |  |  |  |  |  |
| Income deprivation^‡^ (per SD) | | 0.18 | 0.13 | 0.24 | <0.01 |  | 0.08 | 0.03 | 0.13 | <0.01 |
| ^†^Office for National Statistics occupational skills classification.  ^‡^Income deprivation affecting older adults index | | | | | | | | | | |
